# Lipid Peroxidation and Immune Biomarkers Are Associated with Major Depression and Its Phenotypes, Including Treatment-Resistant Depression and Melancholia

**DOI:** 10.1007/s12640-017-9835-5

**Published:** 2017-11-04

**Authors:** Magdalena Sowa-Kućma, Krzysztof Styczeń, Marcin Siwek, Paulina Misztak, Rafał J. Nowak, Dominika Dudek, Janusz K. Rybakowski, Gabriel Nowak, Michael Maes

**Affiliations:** 1 Institute of Pharmacology, Polish Academy of Sciences, Department of Neurobiology, Laboratory of Trace Elements Neurobiology, Smetna Street 12, 31-343 Krakow, Poland; 2 Department of Human Physiology, Institute of Clinical and Experimental Medicine, Medical Faculty, University of Rzeszow, Al. Kopisto Street 2a, 35-959 Rzeszow, Poland; 3 Department of Affective Disorders, Chair of Psychiatry, Jagiellonian University Medical College, Kopernika 21a, 31-501 Krakow, Poland; 4 Chair of Pharmacobiology, Jagiellonian University Medical College, Medyczna 9, 30-688 Krakow, Poland; 5 Department of Drug Management, Jagiellonian University Medical College, Grzegórzecka 20, 31-531 Krakow, Poland; 6 Department of Adult Psychiatry, Poznan University of Medical Sciences, Szpitalna 27/33, 60-572 Poznan, Poland; 7 Department of Psychiatry, Faculty of Medicine, Chulalongkorn University, Bangkok, Thailand; 8 IMPACT Strategic Research Centre, Deakin University, School of Medicine and Barwon Health, Geelong, VIC Australia; 9 Health Sciences Postgraduate Program, State University of Londrina, Londrina, Paraná Brazil; 10 Department of Psychiatry, Medical University of Plovdiv, Plovdiv, Bulgaria; 11 Revitalis, Waalre, the Netherlands

**Keywords:** Major depression, Immune, Inflammation, Cytokines, Melancholia, Oxidative stress

## Abstract

To examine immune-inflammatory and oxidative (I&O) biomarkers in major depression (MDD) and its related phenotypes, we recruited 114 well-phenotyped depressed patients and 50 healthy controls and measured serum levels of interleukin (IL)-1α, soluble IL-1 receptor antagonist (sIL-1RA), soluble IL-2 receptor (sIL-2R), soluble IL-6 receptor (sIL-6R), soluble tumor necrosis factor receptor 60 and 80 kDa (sTNF-R1/R2), and thiobarbituric acid reactive substances (TBARS). Obtained results indicate that MDD is characterized by increased sIL-1RA, sTNF-R1, and TBARS concentrations. Melancholic depression is associated with increased sIL-6R but lowered IL-1α levels. A current episode of depression is accompanied by significantly increased sIL-6R compared to the remitted state. Treatment-resistant depression (TRD) is accompanied by increased sIL-6R and TBARS but lowered sTNF-R2 levels compared to non-TRD patients. These immune markers are not significantly correlated with Hamilton Depression Rating Scale (HDRS), Montgomery-Asberg Depression Scale (MADRS), number episodes, or age at onset. Our findings show that increased sIL-1RA, sTNF-R1, and TBARS levels may be trait markers of depression, while increased sIL-6R levels may be a state marker of melancholia and an acute phase of depression. MDD is accompanied by increased lipid peroxidation and simultaneous activation of immune pathways, and the compensatory anti-inflammatory reflex system (CIRS). TRD is characterized by highly increased oxidative stress and probably increased TNFα and IL-6 trans-signalling. Novel treatments for major depression should target oxidative stress pathways, while new treatments for TRD should primary target lipid peroxidation and also activated immune-inflammatory pathways.

## Introduction

Already in the 1990s, it was shown that major depression (MDD) is accompanied by an immune response and Thelper (Th)1 activation as indicated by elevated serum levels of soluble interleukin (IL)-2 receptor (sIL-2R), increased expression of activation markers on peripheral blood mononuclear cells (PBMCs), e.g., Human Leukocyte Antigen-antigen D Related (HLA-DR) and cluster of differentiation 25 (CD25), resistance of cytokine production by immune cells to dexamethasone administration, and increased stimulated production of interferon (IFN)γ (Maes et al. 1990, 1991, 1992a, 1994; Seidel et al. 1995). Simultaneously, it was shown that major depression is accompanied by a chronic low-grade inflammatory response as indicated by increased levels of macrophage M1-related pro-inflammatory cytokines, including IL-1β and tumor necrosis factor (TNF)α, the soluble IL-1 receptor antagonist (sIL-1RA), increased IL-6 trans-signaling (that is increased IL-6 and sIL-6R levels), and increases in positive acute phase proteins, such as haptoglobin, and complement factors (Maes et al. 1991, 1992b, 1994, 1995a, b; Słuzewska et al. 1995; Frommberger et al. 1997; Mikova et al. 2001). Activation of immune-inflammatory pathways in major depression has now been consolidated in meta-analysis studies indicating increased IL-6, TNFα, sIL-2R, IL-1β, sIL-1RA, and C-reactive protein (CRP) levels in major depression (Howren et al. 2009; Dowlati et al. 2010; Liu et al. 2012; Hiles et al. 2012; Valkanova et al. 2013; Haapakoski et al. 2015; Köhler et al. 2017). These meta-analyses showed additionally that Th2 and Tregulatory (Treg) cytokines (including IL-10, IL4, and IL-5) were not significantly altered in major depression indicating that major depression is characterized by M1 and Th1 activation. There are also some reports that plasma levels of soluble TNF receptor 60 kDa (sTNF-R1) and 80 kDa (sTNF-R2) are elevated in depression (Grassi-Oliveira et al. 2009; Moughrabi et al. 2014; Rizavi et al. 2016). These TNFα receptor subtypes act as decoy receptors by binding sTNFα thereby attenuating TNFα signaling and thus inhibiting TNFα activities (Selinsky et al. 1998; Su et al. 1998).

Other reports showed significant changes in immune-inflammatory signature in clinical subtypes of major depression, most notably major depression with melancholic and atypical features. For example, melancholia is characterized by increased levels of acute phase proteins, e.g., haptoglobin, expression of T cell activation markers and increased resistance of IL-1β and sIL-2R production to dexamethasone administration as compared to simple major depression (Maes et al. 1991, 1992a, 1994). In major depression with atypical features, lowered IL-4 but increased IL-2 concentrations were observed, whereas no changes in IL-6 or TNFα concentrations could be established (Yoon et al. 2012). The lack of M1 activation in atypical depression was further substantiated by findings of Dunjic-Kostic et al. (2013) who showed that IL-6 was significantly increased in melancholic depression but not in atypical depression. These results may suggest that there is a difference in cytokine signature between melancholic and atypical depression the former being associated with M1 and Th1 activation and the latter with a Th1 shift away from Th2 (Maes 2011; Yoon et al. 2012).

Moreover, differences in immune-inflammatory signature were observed in clinical subgroups divided according to staging characteristics, including acute versus remission phase of depression. Thus in vivo, antidepressant treatments, especially with selective serotonin reuptake inhibitors (SSRIs), reduce IL-1β, CRP, and (maybe) IL-6 levels (Maes et al. 1995a; Hannestad et al. 2011; Hiles et al. 2012). Nevertheless, not all papers found a reduction of these immune-inflammatory biomarkers after treatment and could find significant differences between patients in an acute phase of depression and those who were euthymic (Maes 2011), suggesting that increased levels of cytokines (including IL-6 and sIL-6R) are trait markers of major depression. There is also some evidence that treatment-resistant depression (TRD) is characterized by specific immune biomarkers, including increased IL-6, sIL-1RA, and TNFα concentrations (Maes et al. 1997; Lanquillon et al. 2000).

Major depression is accompanied by activated oxidative stress pathways as indicated by increased lipid peroxidation, oxidative damage to DNA and mitochondrial functions, oxidative damage to membranes, including increased expression of neoantigens such as malondialdehyde (MDA) and oxidized low-density lipoprotein (LDL), and lowered antioxidant levels including zinc (review Maes et al. 2011; Liu et al. 2015; Styczeń et al. 2017). There is some evidence that activated immune-inflammatory and oxidative (I&O) pathways are interrelated phenomena in depression (review Moylan et al. 2014). However, there are no data whether increased lipid peroxidation is associated with subtypes of depression, including TRD or melancholia.

This study was performed to examine immune and oxidative biomarkers (namely IL-1α, sIL-1RA, sIL-2R, sIL-6R, sTNF-R1, sTNF-R2, and thiobarbituric acid reactive substances—TBARS), a surrogate marker of MDA production, in major depression and to examine whether these measurements may be state or trait markers of depression, clinical subtypes of depression (including melancholia and atypical depression), or treatment resistance. Here we measure serum concentrations of cytokine receptors (sIL-1RA, sIL-2R, sIL-6R, sTNF-R1, sTNF-R2), rather than their cytokine counterparts (except IL-1α) because measurements of the receptor levels are more reliable, while showing higher levels than cytokines which are often not detectable in peripheral blood (e.g., IL-2). We were also interested in IL-1α levels as many papers in depression focused on IL-1β while neglecting IL-1α (Maes et al. 2012c). Finally, we have measured TBARS as a surrogate biomarker of lipid peroxidation.

## Subjects and Methods

### Subjects

This case-control study included major depressed patients (*n* = 114) and healthy controls (*n* = 50) during the period 21 September 2009 to 30 July 2013. All patients were in- or outpatients in an acute or remission phase of illness and were recruited at the Department of Psychiatry of the University Hospital in Krakow, Krakow, Poland. A psychiatrist made the axis I DSM-IV-TR diagnosis using the semi-structured clinical interview for DSM-IV interview (SCID) axis I disorders (American Psychiatric Association 2000). Diagnosis of melancholic syndrome was based on DSM-IV-TR criteria of major depression episode with melancholic features. The controls were recruited by word of mouth from hospital staff and relatives and friends of hospital staff. In this study, we included male and female subjects of Polish (Caucasian) ethnicity, both sexes and aged 21–70 years. We excluded individuals with (a) axis II personality disorders; (b) psychiatric diagnoses other than major depression, e.g., bipolar disorder, schizophrenia, psychoactive substance dependence (all except nicotine dependence); (c) obesity, that is with a body mass index (BMI) > 30; (d) medical diseases such as primary adrenocortical insufficiency, (auto)immune diseases, renal failure, chronic pancreatitis, primary hypoaldosteronism, carcinomas, hypoparathyroidism, hyperthyroidism, megaloblastic anemia due to iron deficiency, thalasemia, hemochromatosis, liver cirrhosis, Wilson’s disease, nephrotic syndrome and burns; and (e) infections and acute inflammatory responses within a month before the study. We also excluded subjects using nonsteroidal anti-inflammatory drugs, e.g., acetylsalicylic acid, ibuprophen, indomethacin; hydralazine; tetracyclines; fluorochinolones; calcium; iron; chelating agents or glucocorticosteroids; and breast feeding and pregnant women. Healthy controls were excluded for life-time axis I/II diagnoses, including major depression, and for a family history of depression, (hypo)mania, alcohol dependence, and suicidal behaviors. The depressed patients were treated with monotherapy or combinatorial treatments, i.e., SSRIs (*n* = 63), selective noradrenaline reuptake inhibitors (SNRIs) (*n* = 34), tricyclic antidepressants (TCAs) (*n* = 15), mirtazpine (*n* = 5), atypical antipsychotics (n = 15), lithium (*n* = 3), and lamotrigine (*n* = 2). Treatment-resistant depression was defined as the absence of a therapeutic response to at least two further pharmacotherapies with two successively used, different antidepressants at an adequate dose and for an adequate amount of time. The study was approved by the Bioethical Committee of the Jagiellonian University, Krakow, Poland (decision number KBET/77/B/2009; 25.06.2009) and all subjects gave written informed consent prior to participating in this study. The detailed socio-demographic and clinical characteristics of the examined population were presented previously (Styczeń et al. 2015).

### Methods

Severity of depression was measured with the Hamilton Depression Rating Scale (HDRS) (Hamilton 1960) and the Montgomery-Asberg Depression Rating Scale (MADRS) (Montgomery and Asberg 1979). A semi-structured questionnaire was employed to screen for sociodemographic data (age, gender, marital status) and clinical data (age at onset, number of depressive episodes, use of drugs, and nicotine dependence).

Blood (9.8 mL) was sampled around 8.00 a.m. from a brachial vein after an overnight fast. Forty-five minutes later, blood was centrifuged at 1800×*g* for 30 min. Serum was consequently stored at −80 °C until thawed for assay. The cytokine/cytokine receptor biomarkers were determined by enzyme-linked immunoassay (ELISA) using high sensitivity commercially available kits (IL-1α—RayBiotech, Norcross, GA, USA; sIL-1RA, sIL-2R, and sIL-6R—R&D Systems, Minneapolis, MN, USA; sTNF-R1 and sTNF-R2—eBioscience, Hatfield, UK) according to the manufacturers’ protocols. Briefly, appropriate volumes of standards and samples were dispensed into 96-well plates coated with human anti-sIL-1RA, sIL-2R, sIL-6R, sTNF-R1, or sTNF-R2 antibodies and incubated. After extensive washing, an enzyme-linked polyclonal antibody specific for examined proteins was added and incubated. Following a wash to remove any unbound antibody-enzyme reagent, a substrate solution was pipetted to the wells and color develops in proportion to the amount of specific protein bound in the initial step. The reaction was stopped by adding a stop solution, and the absorbance was determined at a wavelength of 450 nm. Lipid peroxidation levels were measured using TBARS Assay kit (Cayman Chemical, Ann Arbor, MI, USA) according to the manufacturer’s recommendations. In this method, MDA reacts with thiobarbituric acid (TBA) under high temperature (90–100 °C) and acidic conditions, generating the MDA-TBA adduct. The MDA-TBA adduct was determined fluorometrically at an excitation wavelength of 530 nm and an emission wavelength of 550 nm. All the measurements were performed using the Synergy 2 multi-mode microplate reader and Gen5 Software (BioTek, Winooski, VT, USA). All assays were performed in duplicate. The detection limits were IL-1α of 0.412 pg/mL, sIL-1RA 18.3 pg/mL, sIL-2R 10 pg/mL, sIL-6R 15.1 pg/mL, sTNF-R1 0.05 ng/mL, sTNF-R2 0.10 ng/mL, and TBARS 0.0625 nmol/mL. The intra- and inter-assay coefficients of variation were < 7.5% for all analytes.

### Statistics

Analyses of variance (ANOVAs) were employed to check differences in sociodemographic, clinical and biomarker data between diagnostic groups, and analyses of contingency tables (Χ^2^ tests) to assess associations between nominal variables. Correlations were calculated using Pearson’s product moment correlation coefficients and stepwise automatic univariate regression analysis. We used automatic stepwise binary regression analysis to delineate the significant explanatory variables predicting major depression (versus controls) or melancholic depression (versus no melancholia) as dependent variables. We employed multivariate general linear model (GLM) analysis with the immune markers as dependent variables and diagnoses as explanatory variables, while adjusting for other background variables including age, sex, and drug status. Consequently, we used tests for between-subjects effects to assess which dependent variables were significantly associated with the significant explanatory variables. Estimated marginal mean values (standard error; SE) obtained by GLM analysis were used to interpret differences among the groups based on protected least significant differences (LSD). Principal component (PC) analysis was used as a data reduction method to create one or two composite factors that reflect use of psychotropic drugs (e.g., SSRIS, SNRIs, TCAs, and atypical antipsychotics), namely the first or first two interpretable PCs generated by PC analysis, whereby the number of PCs is based on eigenvalues > 1. For interpretation purposes, we employed varimax rotation and entered the varimax-rotated PC scores in statistical analyses including multivariate GLM and binary regression analyses as indexes of drug use. We employed Ln transformations of the biomarker data to normalize the data distribution (checked with Kolmogorov-Smirnov test). We used the IBM-SPSS windows version 22 to analyze all data. All tests were two-tailed and a *p* value of 0.05 was used for statistical significance.

## Results

Table 1 shows the sociodemographic, biomarker, and clinical data in healthy controls and depressed patients with and without treatment resistance. There were no significant differences in age, sex ratio, and marital status between the three groups. Nicotine dependence was significantly higher in patients with and without TRD than in normal controls. ANOVA showed significantly higher sIL-1RA levels in depressed patients than in controls and lowered sTNF-R2 levels in depressed patients with TRD than in those without. ANOVA showed that the TBARS levels were significantly different between the three study groups. There were no significant differences in HDRS and MADRS scores, age at onset, number of episodes, and presence of melancholia or atypical depression and a current episode between subjects with and without treatment resistance. We did not employ *p*-corrections to ascertain the results of these multiple ANOVAs/Χ^2^ tests in Table 1 because these results of univariate tests were used to define the explanatory variables that will be employed as determinants of independent association in the ultimate multivariate GLM analyses.

**Table 1:** Sociodemographic, clinical, and biomarker data in patients with treatment-resistant depression (TRD), non-TRD, and healthy controls (HC)

Variables	HC^A^ (*n* = 50)	Non-TRD^B^ (*n* = 72)	TRD^C^ (*n* = 42)	F/O^2^	df	*p*
Age (years)	45.8 (12.4)	48.9 (11.9)	50.2 (8.5)	1.89	2/131	0.155
Gender (F/M)	34/14	42/30	31/11	3.84	2	0.147
Marital status*	18/29	17/55	7/33	5.34	2	0.069
Nicotine dependence (N/Y)	47/3	38/33	21/21	26.76	2	< 0.001
IL-1α (pg/mL)	2.7 (2.4)	2.3 (1.8)	4.9 (10.8)	0.31	2/155	0.732
IL-1RA (ng/mL)	3.30 (2.45)^B, C^	5.30 (3.94) ^A^	5.23 (3.17)^A^	5.61	2/155	0.004
IL-2R (pg/mL)	783 (385)	919 (458)	829 (298)	1.19	2/157	0.308
IL-6R (ng/mL)	26.6 (9.4)	25.8 (6.3)	36.3 (34.8)	2.95	2/150	0.056
sTNF-R1 (ng/mL)	0.18 (0.07)	0.20 (0.006)	0.23 (0.22)	1.71	2/143	0.184
sTNF-R2 (ng/mL)	1.01 (1.32)	0.98 (0.99)^C^	0.84 (1.55)^B^	4.03	2/139	0.020
TBARS (nmol/mL)	2.37 (1.10) ^B,C^	3.18 (1.22)^A, C^	3.86 (1.42)^A,B^	15.34	2/154	< 0.001
HDRS	–	12.2 (8.5)	14.3 (9.7)	1.40	1/112	0.239
MADRS	–	16.5 (13.2)	19.2 (13.7)	1.06	1/112	0.305
Age at onset (years)	–	35.5 (12.7)	37.2 (11.0)	0.53	1/110	0.466
Number episodes	–	5.6 (4.2)	6.0 (4.5)	0.53	1/107	0.612
Melancholia (no/yes)	–	49/22	25/15	0.49	1	0.485
Atypical MDD (no/yes)	–	62/10	29/11	3.13	1	0.087
Current MDD (no/yes)	–	40/29	32/13	2.02	1	0.155

Data are shown as mean (standard deviation; SD). Superscript A, B, C show the significant (*p* = 0.05) pairwise differences among the three study samples

*IL-1α* interleukin-1 alpha, *sIL-1RA* soluble interleukin 1 receptor antagonist, *sIL-2R* soluble interleukin 2 receptor, *sIL-6R* soluble interleukin 6 receptor, *sTNF-R1/R2* soluble tumor necrosis factor receptor 60 kDa/80 kDa, *TBARS* thiobarbituric acid reactive substances

*Marital status: stable relationship + married / single + divorced

**Fig. 1:**
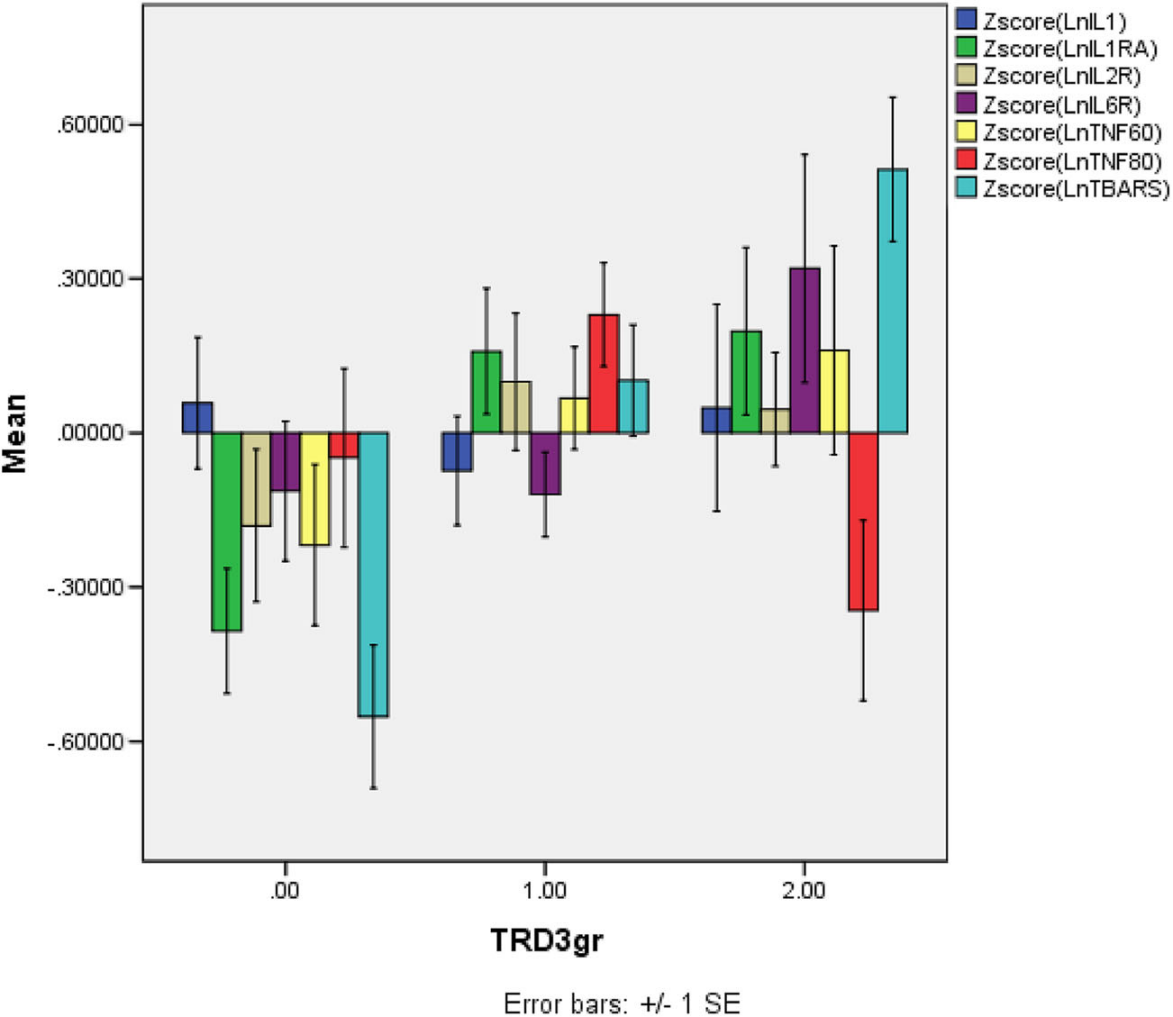
The mean (SE) values of all measurements in healthy controls (0), patients with depression without treatment resistance (1), and those with treatment resistance (2). All data are in z scores computed on the Ln transformation of the measurements. IL-1α, interleukin-1 alpha; IL-1RA, soluble interleukin 1 receptor antagonist; IL-2R, soluble interleukin 2 receptor; IL-6R, soluble interleukin 6 receptor; TNF-60/80, soluble tumor necrosis factor receptor 60 kDa/80 kDa (R1/R2); TBARS, thiobarbituric acid reactive substances

Table 2 shows the results of multivariate GLM analyses performed in the depressed patients only. Immune biomarkers and TBARS data were entered as dependent variables and diagnostic groups (depression with and without TRD) as explanatory variables, while adjusting for age, sex, nicotine dependence, and drug use variables, which were entered as the first two PCs subtracted from four drug state variables. Thus, the first varimax-rotated PC (PC1) explained 43.97% of the variance and loaded highly (> 0.800) on SSRIs (negatively) and SNRIs (positively). The second varimax-rotated PC (PC2) explained 28.3% of the variance and loaded highly (> 0.600) on use of triclyclic antidepressants (negatively) and atypical antipyschotics (positively). We found significant effects of diagnosis, age, and nicotine dependence, but not sex, PC1, and PC2 on the biomarkers. Tests for between-subject effects showed that diagnosis had significant effects on sIL-6R, sTNF-R2, and TBARS levels; age on sTNF-R1 (positive association) and sTNF-R2 (negative association); and nicotine dependence on IL-1α and sIL-2R levels. The second multivariate GLM analysis used the same dependent and explanatory variables but was performed on all subjects and therefore considered the effects of three groups, i.e., controls and depression with and without TRD. We found significant multivariate effects of diagnosis and nicotine dependence but not age, sex, PC1, and PC2. Tests of between-subject effects showed significant associations between diagnosis and sIL-1RA, sIL-6R, sTNF-R1, and TBARS levels. Table 3 shows the adjusted means (after adjusting for age, sex, and drug state) of the biomarkers and results of protected LSDs. sIL-1RA levels were higher in depressed patients without treatment resistance than in controls. sIL-6R levels were higher in patients with treatment resistance than in controls and patients without treatment resistance. sTNF-R2 values were significantly lower in controls and patients with treatment resistance than in patients without treatment resistance. TBARS levels were significantly different between the three subgroups and increased from controls to non-TRD to TRD. Figure 1 shows the mean (SE) values of the z scores of the ln transformed immune measurements.

**Table 2:** Results of multivariate GLM analyses with the six cytokine biomarkers [interleukin (IL)-1α, soluble interleukin 1 receptor antagonist (sIL-1RA), soluble interleukin 2 receptor (sIL-2R), soluble interleukin 6 receptor (sIL-6R), soluble tumor necrosis factor receptor 60 kDa / 80 kDa (sTNF-R1/2)] and thiobarbituric acid reactive substances (TBARS) as dependent variables and diagnostic groups, age, gender, marital status, and nicotine dependence as explanatory variables

Tests	Dependent variables	Explanatory variables	F	df	*p*
(1) Multivariate analysis in MDD patients	7 biomarkers	TRD vs non-TRD Age Sex TUD PC1 PC2	2.33 3.52 0.77 2.76 1.50 0.62	7/64	0.035 0.003 0.619 0.014 0.184 0.735
Between-subject effects	sIL-6R sTNF-R2 TBARS	TRD vs non-TRD TRD vs non-TRD TRD vs non-TRD	5.24 4.66 5.12	1/70	0.025 0.034 0.027
Between-subject effects	sTNF-R1 sTNF-R2	Age (+) Age (−)	7.78 8.86	1/70	0.007 0.004
Between-subject effects	IL-1α sIL-2R	TUD TUD	5.54 0.74	1/70	0.021 0.002
(2) Multivariate analysis in MDD patients and controls	7 biomarkers	TRD, non-TRD, HC Age Sex TUD PC1 PC2	3.31 2.01 1.04 2.71 1.38 0.88	14/208 7/103 7/103 7/103 7/103 7/103	< 0.001 0.061 0.406 0.013 0.224 0.523
Between-subject effects	sIL-1RA sIL-6R sTNF-R2 TBARS	TRD, non-TRD, HC TRD, non-TRD, HC TRD, non-TRD, HC TRD, non-TRD, HC	4.37 3.06 3.27 9.85	2/109	0.015 0.051 0.042 < 0.001

Results of two different multivariate GLM analyses (with tests for between-subject effects) considering the effects of diagnostic groups entered as (1) two groups, i.e., TRD versus non-TRD; or (2) three groups, i.e., TRD versus non-TRD versus healthy controls (HC)

**Table 3:** Marginal means (SE) obtained by GLM analyses in the second multivariate GLM analyses with HC, TRD, and non-TRD as groups

Biomarkers	HC^A^	Non-TRD^B^	TRD^C^
Ln sIL-1RA (ng/mL)	7.83 (0.14)^B^	8.33 (0.11)^A^	8.20 (0.15)
Ln sIL-6R (ng/mL)	10.08 (0.07)^C^	10.11 (0.05)^C^	10.30 (0.07)^A, B^
Ln sTNF-R2 (ng/mL)	− 0.57 (0.14)^B^	− 0.19 (0.10)^A, C^	− 0.52 (0.15)^B^
Ln TBARS (nmol/mL)	0.77 (0.09)^B,C^	1.10 (0.06)^A, C^	1.33 (0.09)^A, B^

^A, B, C^Shows the significant (*p* = 0.05) pairwise differences among the three study samples

Table 4 shows the results of an automatic stepwise regression analysis with depression as dependent variable (and controls as reference group) and all seven biomarkers, age, and sex as explanatory variables. We found three significant biomarkers (Χ^2^ = 31.78, df = 3, *p* < 0.001; Nagelkerke = 0.303) which were positively associated with major depression, i.e., sIL-1RA, TNF-R1, and TBARS levels. Of the subjects, 74.2% were correctly classified with a sensitivity of 75.0% and a specificity of 72.5%.

**Table 4:** Results of automatic logistic regression analyses with major depression (MDD) versus healthy controls as dependent variable and the immune biomarkers, i.e., interleukin-1α, soluble interleukin 1 receptor antagonist (sIL-1RA), sIL-2R, sIL-6R, soluble tumor necrosis factor receptor 60 kDa/80 kDa (sTNF-R1/R2), and thiobarbituric acid reactive substances (TBARS), age, gender, nicotine dependence, education, marital status as explanatory variables

Dependent variables	Explanatory variables	Wald	df	*p*	Odds ratio	lower and upper 95% CI
MDD (versus controls)	sIL-1RA sTNF-R1 TBARS	4.25 4.08 16.39	1 1 1	0.039 0.043 < 0.001	1.83 3.58 7.43	1.03–3.26 1.04–12.37 2.81–19.62

Table 5 shows the results of a multivariate GLM analysis performed on all subjects with the seven biomarkers as dependent variables and with diagnosis, i.e., a current episode versus remission and controls, as explanatory variable, while adjusting for age, sex, nicotine dependence, and drug state as covariates. Tests of between-subject effects show significant effects of diagnosis on sIL-1RA, sIL-6R, and TBARS values. Table 6 shows the adjusted means (after adjusting for age, sex, and drug state) of these three biomarkers and results of protected LSDs. sIL-1RA levels were significantly higher in both depression groups than in controls. sIL-6R values were significantly higher in an acute phase of depression as compared to controls and patients in remission. TBARS levels were significantly higher in both depression groups than in healthy controls. We also examined the associations of the biomarkers and atypical depression by entering diagnosis as three groups, i.e., depression with and without atypical features and controls, in a multivariate GLM analysis (Table 5). We could not find any differences between atypical and non-atypical depression in the biomarkers. There were only significant differences between controls and depressed patients. We also examined the associations of the seven biomarkers with melancholia by entering diagnosis as three groups, namely depression with and without melancholia and controls in a multivariate GLM analysis. However, because we found that the use of psychotropic drugs (PC2) was associated with melancholia, we did not interpret this GLM analysis but examined the characteristics of melancholic depression using a binary logistic regression analysis with melancholia as dependent variable (see Table 7). Table 5 also shows the possible associations between the seven biomarkers and clinical variables, including HDRS and MADRS. There were no significant associations between any of the biomarkers and HDRS and MADRS scores, number of episodes and age at onset of depression.

**Table 5:** Results of GLM analyses with the six immune biomarkers, i.e., interleukin-1α, soluble interleukin 1 receptor antagonist (sIL-1RA), sIL-2R, sIL-6R, soluble tumor necrosis factor receptor 60 kDa/80 kDa (sTNF-R1/R2), and thiobarbituric acid reactive substances (TBARS) as dependent variables and diagnostic groups, age, gender, marital status, and nicotine dependence as explanatory variables

Tests	Dependent variables	Explanatory variables	F	df	*p*
Multivariate analysis in all subjects	7 biomarkers	Current, no-current Age Sex TUD PC1 PC2	2.74 1.76 0.92 2.48 1.59 0.98	14/208 7/103 7/103 7/103 7/103 7/103	0.001 0.103 0.497 0.022 0.148 0.447
Between-subject analyses	sIL-1RA sIL-6R TBARS	HC, current, no-current HC, current, no-current HC, current, no-current	4.19 4.68 8.10	2/109 2/109 2/109	0.018 0.011 0.001
Multivariate analysis in all subjects	7 biomarkers	HC, atypical, no atypical	2.96	14/242	< 0.001
Multivariate analysis in all subjects	7 biomarkers	HC, MEL, no MEL	2.94	14/242	< 0.001
Multivariate analysis in depressed subjects	7 biomarkers	HDRS MADRS Number episodes Age at onset PC1 PC2	0.80 0.49 0.59 1.56 1.53 0.64	7/81 7/81 7/76 7/80 7/65 7/65	0.598 0.840 0.764 0.160 0.174 0.725

HC, current, non-current: diagnostic groups are entered as three groups, healthy controls (HC), current depression, and no-current depression

**Table 6:** Marginal means (SE) obtained by GLM analyses in the second multivariate GLM analyses with HC, current, and no-current as groups

Biomarkers	HC^A^	Current MDD^B^	No current MDD ^C^
Ln sIL-1RA (pg/mL)	7.83 (0.14)^B, C^	8.28 (0.11)^A^	8.33 (0.13)^A^
Ln sIL-6R (pg/mL)	10.08 (0.07)^B^	10.26 (0.05)^A, C^	10.02 (0.07)^B^
Ln TBARS (nmol/mL)	0.77 (0.09)^B, C^	1.23 (0.07)^A^	1.09 (0.09)^A^

^A, B, C^Shows the significant (*p* = 0.05) pairwise differences among the three study samples

**Table 7:** Results of automatic logistic regression analyses with melancholic depression (MEL MDD) versus non-MEL MDD as dependent variable and immune biomarkers, i.e., interleukin-1α, soluble interleukin 1 receptor antagonist (sIL-1RA), sIL-2R, sIL-6R, soluble tumor necrosis factor receptor 60 kDa/80 kDa (sTNF-R1/R2), and thiobarbituric acid reactive substances (TBARS), age, gender, drug state, nicotine dependence, and marital status as explanatory variables

Dependent variables	Explanatory variables	Wald	df	*p*	Odds ratio	lower and upper 95% CI
MEL MDD (versus non-MEL MDD)	IL-1α sIL-6R PC1 PC2	9.52 11.88 2.53 5.72	1 1 1 1	0.002 0.001 0.112 0.017	0.40 29.27 0.68 1.98	0.23–0.72 4.29–199.76 0.43–1.09 1.13–3.47

PC1, PC2: the first two varimax-rotated PC subtracted from the drug state data

**Fig. 2:**
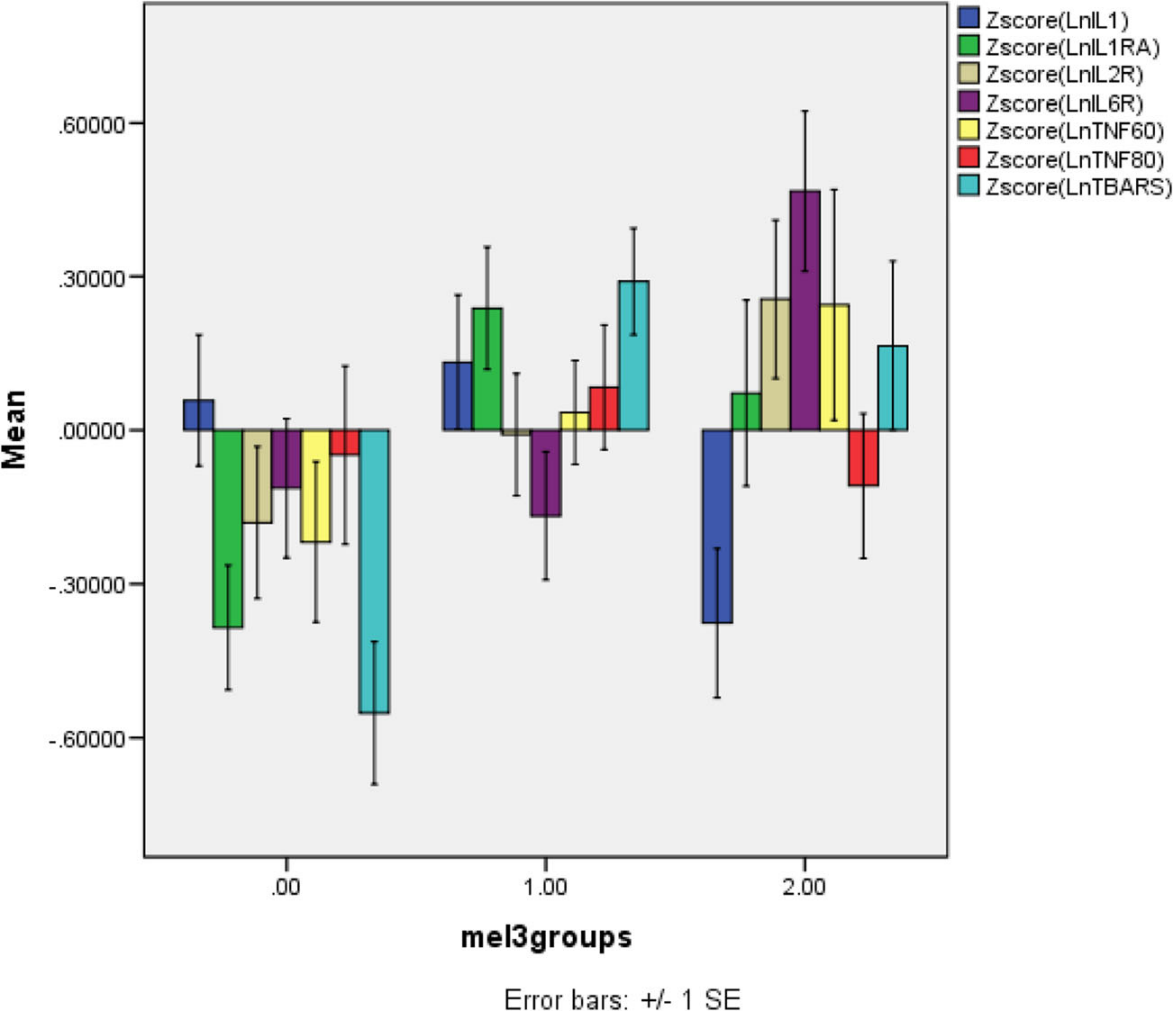
The mean (SE) values of all measurements in normal controls (0), patients with depression without melancholia (1), and those with melancholia (2). All data are in z scores computed on the Ln transformation of the measurements. IL-1α, interleukin-1 alpha; IL-1RA, soluble interleukin 1 receptor antagonist; IL-2R, soluble interleukin 2 receptor; IL-6R, soluble interleukin 6 receptor; TNF-60/80, soluble tumor necrosis factor receptor 60 kDa/80 kDa; TBARS, thiobarbituric acid reactive substances

Table 7 shows the outcome of an automatic stepwise logistic regression analysis with melancholic depression as dependent variable (and non-melancholic depression as reference group) and the seven biomarkers as explanatory variables and with forced entry of PC1 and PC2. Two biomarkers and PC2 were significant in this regression analysis (Χ^2^ = 37.2, df = 4, *p* < 0.001, Nagelkerke = 0.372). IL-1α (inversely) and sIL-6R (positively) were significantly associated with melancholia. The positive association between melancholia and PC2 indicates that more patients with melancholia were treated with atypical antipsychotics. Figure 2 shows the mean (SE) values of the z scores of the ln transformed immune measurements.

The relationships between TBARS and the immune-inflammatory biomarkers were first examined using correlation analyses. Thus, TBARS was significantly correlated with sIL-1RA (*r* = 0.201, *p* = 0.012, *n* = 155) and sIL-6R (*r* = 0.210, *p* = 0.010, *n* = 150), but not IL-1α (*r* = 0.144, *p* = 0.076, *n* = 153), sIL-2R (*r* = −0.058, *p* = 0.470, *n* = 157), sTNF-R1 (*r* = −0.111, *p* = 0.184, *n* = 145), and sTNF-R2 (*r* = −0.134, *p* = 0.114, *n* = 141). Secondly, we used a univariate automatic stepwise regression analysis to examine the correlations between TBARS and the six immune markers, age, sex, TUD, and PC1 and PC2. Of the variance in TBARS, 11.7% (F = 4.40, df = 4/131, *p* = 0.002) was explained by the regression on TNF-R1 (*t* = − 2.20, *p* = 0.028), sIL-1RA (t = + 2.11, *p* = 0.036), TUD (t = + 2.26, *p* = 0.025), and sex (t = + 2.37, *p* = 0.019).

## Discussion

A first major finding of this study is that major depressive disorder is accompanied by increased sIL-1RA, TNF-R1, and TBARS concentrations as compared to normal controls. The sIL-1RA results are in agreement with previous findings showing that depression or depressive phenomenology is accompanied by increased levels of sIL-1RA (Maes et al. 1995b, 2012c; Song et al. 1998; Rief et al. 2001; Ruiz et al. 2007; Milaneschi et al. 2009; Ovaskainen et al. 2009; Lehto et al. 2010; Dahl et al. 2014). mRNA levels of IL-1RA are significantly higher in lymphocytes of depressed patients compared with controls (Rizavi et al. 2016). Meta-analysis studies showed increased sIL-1RA levels in major depression (Howren et al. 2009; Goldsmith et al. 2016). Previous research also showed increased mitogen-stimulated IL-1β production and lymphocyte IL-1β mRNA levels in major depression (Maes et al. 1991; Rizavi et al. 2016). IL-1RA production is stimulated in IL-1 expressing cells (including monocytes, epithelial cells, adipocytes, keratinocytes, and hepatocytes), by IL-1, viral products, some acute phase proteins, and molecular cascades involving IL-6 and IL-4 (Sone et al. 1994; Kay and Calabrese 2004; Perrier et al. 2006). While increased sIL-1RA levels signal immune activation, this protein acts as an endogenous inhibitor of IL-1β and IL-1α activities thereby playing an important role in the resolution of inflammation and tissue repair in IL-1β-associated disorders (Arend and Guthridge 2000). As such, increased levels of sIL-1RA are part of the compensatory (anti)inflammatory reflex system (CIRS), which tends to attenuate an overzealous immune-inflammatory response, including increased IL-1β signaling (Maes et al. 2012a).

Our findings that depression is accompanied by increased sTNF-R1 levels are in agreement with results of Grassi-Oliveira et al. (2009). Moreover, in patients with heart failure, elevated sTNF-R1 levels increase risk of depression (Moorman et al. 2007). Rizavi et al. (2016) detected increased TNF-R1 mRNA levels in lymphocytes of depressed patients as compared with normal volunteers. Increased sTNF-R1 levels were also established in bipolar depression (Barbosa et al. 2011; Teixeira et al. 2015). In fact, sTNF-R1 levels may be higher in bipolar disorder than in major depression (Bai et al. 2015). In our study, we found that non-TRD depression was accompanied by significantly higher levels of sTNF-R2 levels as compared with controls and those with TRD, thus indicating that sTNF-R2 levels may be increased in major depression. Previously, it was shown that depression is accompanied by increased sTNF-R2 levels (Grassi-Oliveira et al. 2009), while sTNF-R2 levels are also elevated in late life depression (Diniz et al. 2010). In patients with stable heart failure, increased sTNF-R2 levels are associated with an increased risk of depression (Moughrabi et al. 2014). In the Rizavi et al. (2016) study, depression was not only accompanied by increased lymphocytic sTNF-R1, but also sTNF-R2 mRNA levels. Increased serum levels of TNFα in major depression were first described by Mikova et al. (2001) and confirmed by a meta-analysis (Liu et al. 2012). The inflammatory effects of TNFα are mediated by cell surface-bound receptors (TNF-R1 and TNF-R2), which may be released in the plasma to exert negative regulatory functions on TNFα signaling (Sedger and McDermott, 2014). Increased levels of both serum soluble receptor subtypes, which act as decoy receptors, may indicate increased negative immune regulation on increased TNFα-mediated inflammatory signaling (Selinsky et al. 1998; Su et al. 1998).

Increased levels of TBARS/MDA have repeatedly been described in patients with depression (Gałecki et al. 2009; Maes et al. 2013; Bajpai et al. 2014), although not all authors were able to find such increases (Magalhães et al. 2012). Nevertheless, a recent meta-analysis (Liu et al. 2015) indicated that serum and red blood cell TBARS/MDA levels are significantly higher in depressed patients than in controls and that antidepressant treatments tended to decrease MDA levels. These findings indicate increased lipid peroxidation and as such the results are in agreement with other reports in depression, including increased levels of oxidized low-density lipoprotein antibodies (Maes et al. 2011). Interestingly, the current study found mild correlations between TBARS levels, on the one hand, and immune biomarkers including sIL-1RA and sIL-6R (both positively) and sTNF-R1 levels (inversely), on the other. Previously, it was reviewed that intertwined activation of oxidative stress and immune-inflammatory pathways take part in the pathophysiology of depression (Maes et al. 2011; Moylan et al. 2014).

The second major finding of this study is that TRD is characterized by increased sIL-6R levels as compared with controls and depressed patients without TRD, lowered sTNF-R2 levels as compared to non-TRD patients and increased TBARS levels as compared with all other study samples. Previously, it was shown that increased levels of IL-6 are associated with TRD (Maes et al. 1997) and that baseline IL-6 production was significantly lower in treatment responders; while IL-6 was significantly higher in nonresponders to antidepressants (Lanquillon et al. 2000). Another study in patients with coronary heart disease and depression was unable to find that increased serum IL-6 levels predict a poor response to treatment with antidepressants (Bot et al. 2011). In an animal model of depression, sustained increases in central nervous system IL-6 may play a pathophysiological role underlying treatment resistance to antidepressants (Sukoff Rizzo et al. 2012). These and other data suggest that IL-6 trans-signaling is associated with treatment resistance in depression (Maes et al. 2014).

Previous studies reported that baseline serum TNFα levels were significantly decreased during treatment with antidepressants in a responder group only (Lanquillon et al. 2000). Other studies, however, were unable to find increased levels of TNFα in depressed patients with TRD as compared to those without TRD (Mikova et al. 2001). In the latter study, there was also a significant association between increased sIL-2R levels and TRD, a finding which could not be replicated in the present study. Moreover, previously no significant association between increased sIL-1RA levels and TRD were found by Maes et al. (1997). Such discrepancies may likely be explained by patient selection criteria. Thus depending on the number of patients suffering from specific depression subtypes (including melancholia and atypical depression), other biomarkers may be found to correlate with TRD.

We found that TRD was accompanied by increased levels of TBARS as compared with non-TRD patients and controls. To the best of our knowledge, this is a first study showing increased TBARS/MDA levels in TRD patients. Previous studies showed increased immunoglobulin (Ig)M responses to MDA in patients with chronic depression and increased IgM responses to a number of MDA-related oxidatively modified membrane markers, including oleic and azelaic acid (Maes et al. 2013).

The third major finding of this study is that melancholic depression was accompanied by significantly increased serum sIL-6R and lowered IL-α levels as compared with non-melancholic major depression. Increased levels of sIL-6R were previously reported in depression and post-traumatic stress disorder comorbid with depression (Maes et al. 1995a, 1999), although the latter studies did not report on a possible correlation with melancholia. Given that IL-6 levels are increased in major depression (Maes et al. 1995a; Słuzewska et al. 1995; Frommberger et al. 1997; Liu et al. 2012), the findings on increased sIL-6R levels in melancholia may suggest that this phenotype is accompanied by increased IL-6 trans-signaling (Maes et al. 2014).

In postmortem brain tissue of depressed patients, increased IL-1α gene expression was detected by Shelton et al. (2011). We are not aware of any other data linking lowered IL-1α serum levels to melancholia, although increased mitogen-stimulated levels of IL-1β were found in melancholic depression (Maes et al. 1991). While IL-1α usually functions as an autocrine growth factor, which only reaches the environment following cellular necrosis, IL-1β is secreted from activated immune cells and plays a major role in the initiation and maintenance of inflammatory responses (Maes et al. 2012c; Dinarello 2011). Therefore, it is not clear whether the current results on IL-1∀ may have clinical relevance. The present study did not show significant associations between atypical depression and any of the biomarkers. These negative findings extent those of previous papers showing no significant associations in serum IL-6 and TNFα concentrations, although other cytokines (not measured in our study) may be altered in atypical depression (Yoon et al. 2012).

The fourth major finding of this study is that an acute episode of major depression is characterized by increased sIL-6R levels as compared with the remitted state. Given that an acute episode of depression is also accompanied by increased serum IL-6 levels (Maes et al. 1995a; Liu et al. 2012), the results suggest increased IL-6 trans-signaling occurs in acute depression. Not all studies, however, were able to find increased serum sIL-6R levels (Rief et al. 2001) or lymphocyte IL-6R mRNA expression (Rizavi et al. 2016) in depression. It should be added that we did not find significant associations between the biomarkers and illness severity. Also, no significant associations could be found between any of the biomarkers and number of depressive episodes. Previous research showed that sensitization may occur in some selected immune biomarkers, including TNFα, IL-6, IL-1 (α + β), and sIL-1RA levels. Thus, the number of episodes was significantly associated with increasing levels of TNFα and IL-1 (Maes et al. 2012b), while greater increases in serum sIL-1RA and IL-6 were established in puerperal women who had suffered from a lifetime depression (Maes et al. 2001).

The results of the current study should be interpreted with regards to its limitations and strengths. Firstly, this is a case-control study and therefore no causal inferences may be made. Secondly, it would have been more informative if we had also measured serum IL-6 and IL-1β and other cytokine levels, including Th17, T regulatory, and Th2 cytokines. Thirdly, included were individuals between 21 and 70 years old and, consequently, our results cannot be generalized to older or younger populations. Strengths are that we included relevant phenotypes of major depression, while adjusting our data for background variables including age, sex, smoking, and the drug state of the patients.

In conclusion, major depression is characterized by increased sIL-1RA, sTNF-R1, and TBARS concentrations, while the acute phase of depression is accompanied by higher sIL-6R levels (compared with the remitted state) and melancholia by higher sIL-6R but lower IL-1α levels. As such, increased sIL-1RA, sTNF-R1, and TBARS levels may be trait markers of major depression, while increased sIL-6R levels may be state markers of melancholia and an acute episode. These findings show that depression, and especially acute depression and melancholia, are associated with increased lipid peroxidation and immune alterations pointing towards immune activation (increased sIL-6R), increased activity of the CIRS (increased sIL-1RA), and signs of immunosuppression (lowered IL-1α and increased TNF-R1/R2 levels). TRD is accompanied by increased TBARS and sIL-6R but lowered sTNF-R2 levels, suggesting that highly increased lipid peroxidation, immune activation, and lowered immunosuppression increase risk towards TRD.

As discussed previously, the CIRS response has anti-inflammatory and negative immunoregulatory effects thereby protecting against immune-inflammatory responses (Maes et al. 2012a, 2016). Previously, we have discussed that targeting immune-inflammatory pathways in depression by anti-inflammatory drugs is probably not the best treatment strategy. Indeed, interfering with the equilibrium between protective (CIRS) and more detrimental (increased neurotoxicity by inflammatory cytokines) forces may have many unwanted or unpredictable consequences (Maes et al. 2016). More precise knowledge on the immune-inflammatory response in relation to the CIRS in depression and the impact of the new drugs on both systems is needed before using drugs that interfere with their equilibrium. Moreover, lipid peroxidation is—by far—a more important trait marker of depression and TRD as compared with the immune biomarkers, suggesting that increased oxidative stress in the primary target. Finally, since TRD is characterized by increased TBARS and sIL-6R and lowered sTNF-R2 levels, we may suggest that increased lipid peroxidation coupled with possible signs of increased TNFα and IL-6 trans-signaling may play a role in TRD. Therefore, multi-targeting immune-inflammatory and oxidative stress pathways may have therapeutic promise (Maes et al. 2016).
